# Effectiveness of the Wellness Together Canada Portal as a Digital Mental Health Intervention in Canada: Protocol for a Randomized Controlled Trial

**DOI:** 10.2196/48703

**Published:** 2024-01-30

**Authors:** Syaron Basnet, Michael Chaiton

**Affiliations:** 1 Centre For Addiction and Mental Health University of Toronto Toronto, ON Canada; 2 Dalla Lana School of Public Health University of Toronto Toronto, ON Canada

**Keywords:** Wellness Together Portal, randomized trial, COVID-19, mental health, digital health, digital intervention, substance use, portal, effectiveness, well-being

## Abstract

**Background:**

The Wellness Together Canada (WTC) portal is a digital mental health intervention that was developed in response to an unprecedented rise in mental health and substance use concerns due to the COVID-19 pandemic, with funding from the Government of Canada. It is a mental health and substance use website to support people across Canada providing digital interventions and services at no cost. Two million people have visited the WTC portal over the course of 1 year since launching; however, rigorous evaluation of this potential solution to access to mental health care during and after the COVID-19 pandemic is urgently required.

**Objective:**

This study aims to better understand the effectiveness of the existing digital interventions in improving population mental health in Canada.

**Methods:**

The Let’s Act on Mental Health study is designed as a longitudinal fully remote, equally randomized (1:1), double-blind, alternative intervention–controlled, parallel-group randomized controlled trial to be conducted between October 2023 and April 2024 with a prospective follow-up study period of 26 weeks. This trial will evaluate whether a digital intervention such as the WTC improves population mental health trajectories over time.

**Results:**

The study was approved by the research ethics board of CAMH (Centre for Addiction and Mental Health, Toronto, Ontario, Canada). It is ongoing and participant recruitment is underway. As of August 2023, a total of 453 participants in the age group of 18-72 years have participated, of whom 70% (n=359) are female.

**Conclusions:**

This initiative provides a unique opportunity to match people’s specific unmet mental health and substance use needs to evidence-based digital interventions.

## Introduction

Mental health problems are the leading cause of global disability affecting worldwide populations including Canadians. The Bell Let’s Talk campaign promotes mental health awareness by engaging Canadians in encouraging people to speak up about mental health. Yet, many Canadians lack access to mental health care services, with equity in access being a key concern for specific groups (eg, gender, race and ethnicity, socioeconomic status, geographic isolation, and immigration status). As a result of the COVID-19 pandemic and preventive measures such as social distancing, an “echo pandemic” of declining mental health in the Canadian population has emerged [1]. The COVID-19 pandemic is exacerbating mental health challenges and widening service gaps, especially among those in marginalized groups and those with preexisting mental health issues [2,3]. Research on mental health during the pandemic has found that 40% of Canadians stated that their mental health has declined due to the COVID-19 pandemic, and 48% of individuals felt anxious or worried [4,5]. Even prior to the pandemic, the burden of mental illness was widespread in the population. Worldwide, an estimated 264 million people are affected by depression alone [6]. Mood and anxiety disorders are the most prevalent mental health issues in Canada, occurring among 11.6% of Canadian adults [7]. Our mental health care system is overwhelmed and too underfunded to address the pervasiveness of mental health challenges [8]. At the same time, many people are reluctant to maintain or improve their mental health, with a stigma-based perception that mental health is only an issue for people with a diagnosed mental disorder, rather than being a concern for everyone [9]. The experience of the COVID-19 pandemic demonstrates that it is critical to provide tools to all Canadians, which will improve mental health and provide self-assessment and self-help aids at the population level [10].

The digital intervention for mental health, the Wellness Together Canada (WTC) portal [11], was developed in response to an unprecedented rise in mental health and substance use concerns due to the COVID-19 pandemic, with funding from the Government of Canada. It is a mental health and substance use website to support people across Canada, providing digital interventions and services at no cost. It partners with other digital interventions to be the home portal for access to digital interventions from 1:1 support, peer groups, and self-help tools. WTC is a one-stop, web-based portal, available nationally, which provides all people in Canada with 24/7 free and confidential access to a web-based network of information and psychosocial support services, which comprise triage and self-monitoring tools, mental health promotion information, self-guided apps, coached and peer support programs, mental health counseling, and crisis intervention. Services are readily accessible, available in French and English, and are made available through a variety of digital media (eg, computer and phone) and web-based services (eg, voice, text, web-based chat, and responsive web design). The portal is stigma-free and recovery-oriented, providing a wide range of intervention options, and follows a continuous improvement process based on stakeholder engagement, program data, and user feedback. Two million people have visited the WTC portal over the course of 1 year since launching; however, rigorous evaluation of this potential solution to access to mental health care during and after the COVID-19 pandemic is urgently required.

This initiative provides a unique opportunity to match people’s specific unmet mental health and substance use needs to evidence-based digital interventions. Canadians will have a tool that provides valuable information, self-assessment on mental health, access to personalized digital interventions, and an avenue for everyone to participate in better understanding and improving mental health in Canada.

This study aims to evaluate the effectiveness and cost-effectiveness of the WTC portal by conducting a randomized controlled trial. The control group will be referred to the usual care tools available on the CAMH (Centre for Addiction and Mental Health) website. CAMH provides a range of self-care interventions, particularly different mental health programs and resources such as “Game Changers: Self-care information,” which is a hub of resources to help start a conversation about health, including a self-help booklet series, a relaxation guide, and free web-based education with a personalized dashboard, which provides an opportunity for the client to self-monitor (symptoms or physiological processes), build awareness, and improve self-efficacy and visualization. Usual care tools such as digital interventions have a self-care and self-help component. Many studies have shown positive outcomes deemed acceptable and feasible with >95% completion rates and a high degree of participation with self-care interventions; some offer significant early benefits of tailored self-care programs [12,13]. A meta-analysis indicates that these interventions can be effective and potentially be future home-based interventions, especially in mental health care such as digital interventions; hence, it has been chosen as a control intervention in this study.

## Methods

### Trial Design

**Figure 1:**
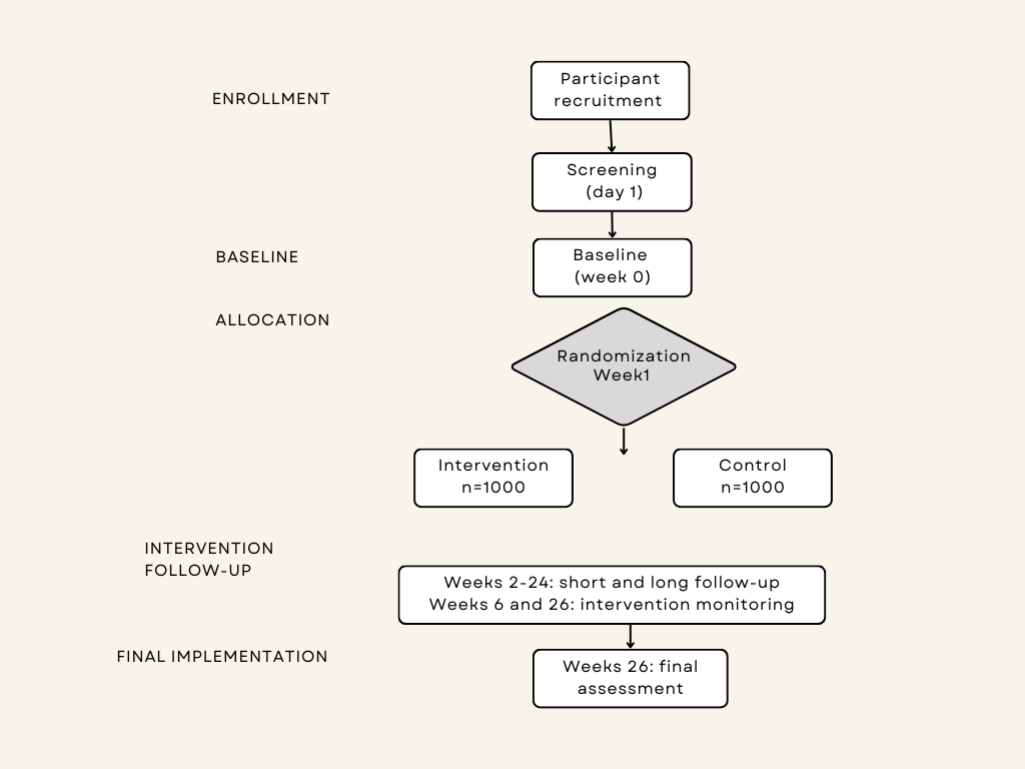
Flow chart showing participation stages and assessments in the Let’s Act on Mental Health Survey of 2022.

The Let’s Act on Mental Health study is a longitudinal, fully remote, equally randomized (1:1), double-blind, alternative intervention–controlled, parallel-group randomized controlled trial to be conducted between October 2023 and April 2024 with a prospective follow-up for 26 weeks. This trial will evaluate whether digital interventions such as WTC improve population mental health trajectories over time. The trial will consist of 1 baseline assessment at week 0, 13 biweekly short and long follow-ups from week 24, intervention monitoring twice at weeks 6 and 26, and 1 final assessment at the end of the 26-week follow-up period. An overview of the trial process is shown in Figure 1.

The study’s overall objective is to evaluate the effectiveness and cost-effectiveness of the WTC portal by conducting a randomized controlled trial. We will use a “Knowledge-to-Action Framework” [14] with extensive stakeholder and cross-sectoral engagement and will incorporate program elements with people with lived experience.

The research questions we seek to answer with this study are the following: (1) how effective is the WTC portal in altering the trajectories of mental health and substance use, primarily anxiety, compared to usual care? (2) What is the benefit of WTC versus usual care in terms of incremental costs, health outcomes, ongoing recruitment, and maintenance costs compared to the cost per quality-adjusted life year (QALY) gained using these interventions? (3) What is the differential impact of WTC by age, sex or gender, race, and socioeconomic status?

### Participants and Recruitment

Adults (N=2000) will be recruited using both paid and free web-based advertisements (social networking sites, Google advertisements, Facebook, Instagram stories, Twitter, etc). The Facebook business account of CAMH will be used to advertise the study and recruit participants. In the case of web-based advertisements, participants will click on a link in the advertisement that will direct them to a REDCap (Research Electronic Data Capture) survey, which will screen them for eligibility (see Textbox 1 for inclusion and exclusion criteria). The project’s social media pages and CAMH’s page will be used for advertisements. Further, we will also approach the participants from other ongoing studies at CAMH including the Vaping Research Panel, Smoking Panel, and those who had agreed to be contacted for future studies. We will not cold contact previous participants; we will ensure that initial contact with the participants is made by the research staff who had previously contacted and are known to them. This will help establish trust and compliance for the Let’s Act on Mental Health study.

**Inclusion and exclusion criteria for the study.**

**Inclusion criteria:**

- Adults (≥18 years of age) residing in Canada and speaking English or French
- Ability to complete study assessments in English
- Interest in the use of digital interventions to improve their mental health
- Having internet access

**Exclusion criteria:**

- People who are at risk for suicide (based on the 9-item Patient Health Questionnaire scores)

### Inclusion Criteria

Inclusion criteria are (1) being aged 18 years or older, (2) residing in Canada and speaking either English or French, (3) able to complete study measures in English, (4) willing or interested to use the digital interventions to improve their mental health, and (5) having internet access. Furthermore, concomitant psychotropic medication use will be allowed.

### Exclusion Criteria

The main exclusion criterion was being at risk for suicide (based on the 9-item Patient Health Questionnaire [PHQ-9] score).

Participants who are not eligible for this study after screening will be redirected to a different page, informing them that they are not eligible and thanking them for their time.

### Participant Withdrawal Criteria

Participants will not be withdrawn from the study for not wanting to receive the WTC or control intervention. Participants’ intention to use and the reason to not use the intervention will be identified through self-assessments. However, they will be withdrawn from the study if suicidal thoughts occur in any of the long-term follow-up assessments at weeks 4, 8, 12, 16, 20, and 24.

### Sample Size

The target sample size for the population survey and learning systems sample is 2000. This sample size will also allow for the estimation of the impact of the dashboard and app recommendations on score trajectories of the PHQ-9 and 7-item Generalized Anxiety Disorder Scale (GAD-7). Based on an estimated effect size of Cohen *d*=0.17 for changes in the PHQ-9 score and Cohen *d*=0.13 for changes in the GAD-7 score (based on the expected effects of the dashboard) [15], an expected dropout rate of no more than 35% [16], and population mean and SD estimates of PHQ-9 and GAD-7 scores [17,18], we estimate having a power of 0.90 and a type I error rate of 0.05 for detecting improvements in PHQ-9 and GAD-7 scores for subsamples with at least 1000 participants. Therefore, in this study, we will have a sample size of a total 2000 participants, with 1000 randomized to each group.

### Randomization

At week 1, after baseline assessments, participants will be randomly allocated through email invitation to intervention or control groups using a simple randomization method equivalent of tossing a coin to ensure double-blinding. Participants deemed eligible after a manual-fraud check will be emailed a personalized URL invitation based on the groups in the study by the research personnel within the study, using the email template attached.

### Blinding

Both participants and the research team involved in the trial will be blinded about the groups. The groups will be observed in an identical manner by the study team to ensure randomization. Further, to ensure that the study team is double-blinded, the potential participant receiving the intervention or usual care will be contacted by a research assistant outside the research team. The 2 groups will be followed up for 26 weeks to determine whether there are any differences in outcomes between them. The results and subsequent analysis of the trial will be used to assess the effectiveness of the WTC portal, which is the extent to which the service benefits the patients rather than doing them any harm.

### Intervention

The intervention group will be invited to the WTC portal, which provides options for digital interventions. A single-sign on system used by WTC will be used to facilitate ease of transfer.

### Control

The control group will be referred to the usual care tools available on the CAMH website, particularly (different mental health programs and resources such as “Game Changers: Self-care information,” which is a hub of resources to help start conversations about health, self-help booklet series, relaxation guides, and free web-based education with a personalized dashboard), which provides an opportunity to clients for self-monitoring (symptoms or physiological processes), build awareness, improve self-efficacy, and visualization. The control group will not receive process monitoring assessments at week 6 or 26. Service usage and contamination in the control group will not be monitored.

### Duration and Assessments

The expected duration of each participant’s involvement in the study is for 6 months with baseline, weekly short follow-ups, intervention monitoring, and final assessments. Participants from the intervention and control groups will be asked to fill 1 baseline assessment at week 0 and 12 biweekly follow-ups from week 2 until week 24 including process monitoring of the intervention at weeks 6 and 26. Lastly, one final assessment will be carried out at week 26, for a total duration of 6 months. Hence, in total, a participant will complete 14 assessments. Participants will be sent weekly reminder emails to complete the assessments for compliance. The study team will track people who are lost to follow-up by communicating with them regularly through weekly emails and will continue to do so until the end of the study. They will be sent emails every week and will be provided an opportunity to complete the assessments when they show up until the end.

### Ethical Considerations

This study is approved by the Research Ethics Board (REB) of CAMH (reference <REB#: 037-2022>, version 7.0). The protocol will be reapproved by the REB if it requires amendment. Participants will be screened and provided a unique ID number to protect their personal information. An electronic informed consent form has been prepared in REDCap to remotely obtain consent from the participants prior to enrolling them in the trial. A REDCap survey has also been designed separately for participants’ eligibility screening at baseline, biweekly follow-ups, intervention monitoring, and final assessment over the 26-week period. Participants will be allowed to withdraw at any time without penalty.

Participants who meet the eligibility requirements of the initial screening form will be prompted to fill out a contact form with their first name and email address. Participants deemed eligible after a manual fraud check will be emailed a personalized URL to the baseline questionnaire by the research staff and those who are deemed ineligible by the fraud check will be sent a notification from REDCap about their ineligibility. The baseline questionnaire is largely composed of questions about age, sex, gender, race and ethnicity, education, and 3 digits of the participants’ postal code to later connect it with census data and community-level equity data. Lifetime history of substance use and mental health will be ascertained, including problematic alcohol use (assessed using Alcohol Use Disorders Identification Test item 3 about how often they had 6 or more drinks on 1 occasion in the past year) and drug use (assessed using the modified Drug Use Disorders Identification Test). Finally, we will measure time to completion for data quality reasons, but this variable will not be visible to participants.

This randomized population trial will be compensating thousands of participants (N=2000). We will compensate participants at a rate sufficient to motivate them to comply with the study protocol and to complete all surveys and measures. This will include a CAD $10 (US $7.24) Giftbit [19] e-card after completing the initial baseline survey at week 0, and then CAD $24 (US $17.38) for completing the 12 assessments during biweekly follow-up in weeks 2-24, and finally CAD $10 (US $7.24) for final assessments at week 26. The follow-up payment will be based on completion rate, which, in turn, is based on the number of assessments completed at a rate of CAD $2 (US $1.45) biweekly. Hence, a participant completing 100% of the assessments (N=14) will receive CAD $44 (US $31.87) in total.

### Privacy and Confidentiality

The master linking log will be stored in a separate folder than the other data files on the CAMH server. The master linking log will be used to identify contact information in order to send the baseline questionnaire link, follow-up questionnaire, and gift cards; to store status of interest in the qualitative study and future research studies; and to monitor completion of baseline and follow-up surveys. A numerical participant ID number will be used to differentiate participants in an anonymous manner across measures and follow-up questionnaires. 

Participants who complete surveys will receive e-gift cards through Giftbit, which allows one to select a gift card of one’s choice. Giftbit Incentives is a secure platform that allows bulk and automated sending of e-gift cards, and has been used by the principal investigator (MC) in previous studies at CAMH. Participant information shared with Giftbit will be limited to their first name and email address, in order to allow personalized e-gift card emails. 

### Safety and Adverse Events

Since the study procedures are not at higher than minimal risk, adverse events (AEs) and serious AEs (SAEs) are not expected. If any unanticipated problems related to the research involving risks to participants or others occur during this study, they will be reported to applicable stakeholders in alignment with institutional policies. AEs that are not serious but are notable and could involve risks to participants will be documented in accordance with institutional documentation requirements.

### Outcome Measures

#### Primary Outcomes

The primary outcomes are the changes in trajectories of mental health, primarily anxiety at baseline (week 0) until final assessments (week 26) between 6 months after invitation to the WTC intervention or usual care. The trajectories are also measured biweekly with short follow-ups at different time points from week 2 until week 26. We will use multilevel modeling to describe within-person differences in anxiety and depression at different time points. We will compare the observed trajectory with those of the control group. Interactions by demographic subgroups (primarily age, gender, race, and socioeconomic status) will be assessed for identification of potential effect modifications. 

#### Secondary Outcomes

An economic analysis will be conducted by comparing incremental costs and health outcomes for the Wellbeing Tracker and WTC compared to within-person expected trajectories using a cost-effectiveness acceptability curve. These costs will be based on the costs associated with the ongoing recruitment and maintenance costs of WTC compared to the cost per QALY gained through the use of these interventions [20]. To be somewhat comparable with World Health Organization’s standards for ranking public health interventions, the determination of whether the WTC overall is highly cost-effective will be based on a threshold of CAD $124 (~US $100) per QALY gained [21]. QALYs will be estimated using the SF-6D (Short-Form Six-Dimension), calculated on the basis of the SF-12 (12-item Short Form Survey) [21]. This analysis will provide an empirical basis for the effect of allocation of health care resources in Canada. To aid other countries, we will also base cost-effectiveness calculations based on estimations that incorporate implementation costs of the Wellbeing Tracker and WTC. We will also estimate cost-effectiveness based on clinically Reliable Change Index scores (ie, 5 points on the PHQ-9 [22] and a 6-point change on the GAD-7 [23], as well as employment status. The schedule of the Let’s Act on Mental Health study is outlined in Table 1.

**Table 1:** Schedule of the Let’s Act on Mental Health Survey of 2022.

Procedures	Study period (day 1 to week 26)						
Enrollment	Day 1	Week 0	Week 1	Weeks 2 and 4	Week 6	Weeks 8-24	Week 26
Eligibility screening	✓						
Informed consent	✓						
Screening assessments (PHQ-9^a^ item 9)	✓						
Drug use (DUDIT-C modified^b^)	✓						✓
Problematic alcohol use (AUDIT-C^c^)	✓						✓
Enrollment							
Demographics		✓					
3-Digit postal codes		✓					
Baseline with self-reported questionnaires (GAD-7^d^, PHQ-4^e^, Current Substance Use, SF-12^f^, and WSAS^g^)		✓					
Randomization to the WTC^h^ intervention or usual care group through an email invitation			✓				
Biweekly short and long follow-up (GAD-7, PHQ-4, Current Substance Use, SF-12, and WSAS)				✓		✓	
WTC and usual care monitoring for contamination and compliance checks					✓		✓
Final assessment (GAD-7, PHQ-4, current substance use, SF-12, and WSAS)							✓

^a^PHQ-9: 9-item Patient Health Questionnaire.

^b^DUDIT-C modified: modified Drug Use Disorders Identification Test.

^c^AUDIT-C: Alcohol Use Disorders Identification Test.

^d^GAD-7: 7-item Generalized Anxiety Disorder Scale.

^e^PHQ-4: 4-item Patient Health Questionnaire.

^f^SF-12: 12-item Short Form Survey.

^g^WSAS: Work and Social Adjustment Scale.

^h^WTC: Wellness Together Canada.

### Data Management and Availability

All data will be collected and managed in the REDCap electronic case report forms. This system is maintained on central CAMH servers, with data backed up daily, and is supported by the Research Informatics department. This will strengthen the data’s accuracy and maintain data quality. Only authorized personnel will monitor the completion of surveys, sending up to 3 reminder emails to participants who have not completed baseline surveys, up to 3 reminder emails to participants who have not completed follow-up surveys, and up to 3 reminder emails to participants who have not completed 6-month surveys.

The data sets will be stored on the secure CAMH server, and only for the minimum amount of time needed for fraud screening. Only trained research staff will have access to the data sets, and they will only use the merged data set for fraud screening purposes. As participants are screened, they will be entered into the screening log. Those deemed eligible will have their contact information, study ID number, and first name copied by research staff into the master linking log (a separate Excel [Microsoft Corp] file stored in a separate location from the fraud screening data). Once each round of fraud screening is completed, the merged data file will be securely destroyed. The original contact files may remain on the CAMH server to cross-check study IDs with the screening log, in the event that an ineligible individual contacts staff and inquiries about their eligibility. 

### Statistical Analysis

A statistician who is not affiliated with this study will perform the statistical analyses for Linear random effects modeling—which accounts for repeated measures over time—that will be used to study the intervention’s effect [24]. We will also use multilevel modeling to describe within-person differences in anxiety and depression. We will then compare this observed trajectory with the control group’s trajectories.

The level of change in anxiety from weeks 0-26 (baseline to final assessment) over the 6 months’ time period is the primary trajectory of concern for mental health in this study. We will test the hypothesis that the trajectories are changing over time and the intervention’s effects are expected to be observed as changes in self-reported mood, which will be measured with the mood scores, cutoff scores, sensitivity, etc, obtained at various time points. Interactions by demographic subgroups (primarily age, gender, race, and socioeconomic status) will be assessed for identifying potential effect modifications.

Analysis will be based on the intention-to-treat principle, as this method allows to draw an unbiased conclusion regarding the effectiveness of an intervention. It also preserves the benefits of randomization, which cannot be assumed when using other methods of analysis [25]. Further, this method is also more effective in accommodating missing data, as missingness is assumed at random.

## Results

The study is ongoing and the first wave data collection is complete; we are analyzing data from the baseline and weeks 2-26. The study is ongoing at week 26, we have analyzed the preliminary baseline data and weeks 2 and 4 short and long follow-ups to understand the ongoing trajectory of depression. Until now, 453 individuals in the age group of 18-72 years have responded to the study invitation emails.

The trial is currently in the ethics approval process. Thus far, ethics approval has been granted by the REB of CAMH, and participant recruitment is ongoing. The study will be conducted in accordance with the REB-approved study documents and the determinations (including any limitations) of the REB, and in compliance with the REB’s requirements.

## Discussion

### Anticipated Findings

At baseline, with regard to gender and race, a greater proportion of participants are female (n=359, 75.57%) and White (n=335, 67.95%). We aim to generalize this study to the Canadian population; hence, we have been weighting samples at the census level based on gender and province to make this study representative. Weighing of the sample will help us understand how generalizable the sample and advertisements are and whether they are adequately representative. The SF-12, measuring mental and physical health, has revealed a significant decline in mental health from baseline to short and long follow-ups in this study. It is too early to discuss how this will develop longitudinally, but the preliminary results suggest a larger decline in mental health than in physical health in the Canadian population.

### Limitations

The foreseeable factors that could compromise the outcome of this study is the monitoring of WTC usage, which is based on self-reports. However, to mitigate the challenge, the study team will ensure detailed monitoring and contamination of intervention at 1 and 6 months. The study team will continuously explore its potential partners for incorporating artificial intelligence within the digital interventions, such as the WTC, for real-time monitoring of service usage. Service usage and contamination in the control group will not be evaluated.

Digital health interventions for delivering mental health care were relevant even before the COVID-19 pandemic. After the pandemic, it has proven to be a safer alternative to face-to-face treatment [26]. It offers great potential, and there is an urgent need for up-to-date information on the mental health impacts post the COVID-19 pandemic. Especially after pandemic, there is a dire need of population-level mental health interventions to improve access to and the quality of mental health care. The challenges of mental health care are its reach at the population level. Interventions such as WTC have shown great potential in developing interest toward mental health interventions in any population; however, the challenge of consistency remains to be studied.

## Abbreviations

AE: adverse event
CAMH: Centre for Addiction and Mental Health
GAD-7: 7-item Generalized Anxiety Disorder Scale
PHQ-9: 9-item Patient Health Questionnaire
QALY: quality-adjusted life year
REB: Research Ethics Board
REDCap: Research Electronic Data Capture
SAE: serious adverse event
SF-6D: Short-Form Six-Dimension
SF-12: 12-item Short Form Survey
WTC: Wellness Together Canada
